# Modeling the Effects of IL-1β-mediated Inflammation During Ex Vivo Lung Perfusion Using a Split Human Donor Model

**DOI:** 10.1097/TP.0000000000004613

**Published:** 2023-05-05

**Authors:** Thomas Pither, Lu Wang, Lucy Bates, Morvern Morrison, Catriona Charlton, Chelsea Griffiths, Jamie Macdonald, Venetia Bigley, Maria Mavridou, Joseph Barsby, Lee Borthwick, John Dark, William Scott, Simi Ali, Andrew J. Fisher

**Affiliations:** 1 Regenerative Medicine, Stem Cells and Transplantation Research Group, Faculty of Medical Sciences, Translational and Clinical Research Institute, Newcastle University, Newcastle upon Tyne, United Kingdom.; 2 Institute of Transplantation, Freeman Hospital, Newcastle upon Tyne Hospitals NHS Foundation Trust, Newcastle upon Tyne, United Kingdom.

## Abstract

**Background.:**

The association between interleukin-1β (IL-1β) concentrations during ex vivo lung perfusion (EVLP) with donor organ quality and post-lung transplant outcome has been demonstrated in several studies. The mechanism underlying IL-1β-mediated donor lung injury was investigated using a paired single-lung EVLP model.

**Methods.:**

Human lung pairs were dissected into individual lungs and perfused on identical separate EVLP circuits, with one lung from each pair receiving a bolus of IL-1β. Fluorescently labeled human neutrophils isolated from a healthy volunteer were infused into both circuits and quantified in perfusate at regular timepoints. Perfusates and tissues were subsequently analyzed, with perfusates also used in functional assays.

**Results.:**

Neutrophil numbers were significantly lower in perfusate samples collected from the IL-1β-stimulated lungs consistent with increased neutrophil adhesion (*P* = 0.042). Stimulated lungs gained significantly more weight than controls (*P* = 0.046), which correlated with soluble intercellular adhesion molecule-1 (R^2^ = 0.71, *P* = 0.0043) and von-Willebrand factor (R^2^ = 0.39, *P* = 0.040) in perfusate. RNA expression patterns for inflammatory genes were differentially regulated via IL-1β. Blockade of IL-1β significantly reduced neutrophil adhesion in vitro (*P* = 0.025).

**Conclusion.:**

These data illustrate the proinflammatory functions of IL-1β in the context of EVLP, suggesting this pathway may be susceptible to therapeutic modulation before transplantation.

## Graphical abstract

**Figure:**
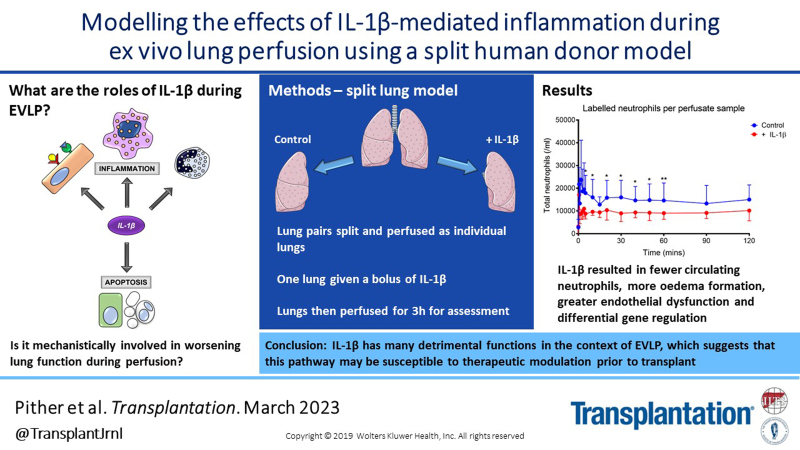

## INTRODUCTION

There remains an ever-expanding disparity between the number of donor lungs deemed suitable for transplantation and the number of individuals on the transplant waiting list.^1^ Despite efforts to expand the donor lung pool, a substantial proportion of donor lungs offered for transplantation are judged unsuitable for transplant. This perceived lack of suitability is primarily due to the potential higher risk of adverse outcomes, such as primary graft dysfunction (PGD). Thus, there is a major clinical need to increase rates of donor lung utilization without increasing adverse posttransplant outcomes.

Since ex vivo lung perfusion (EVLP) was piloted by Steen et al in Lund, Sweden, in 2001 as a decision-making assessment tool,^2^ it has progressed to being considered a powerful therapeutic platform for prolonged preservation and reconditioning.^3-5^ Commercial EVLP devices have been used in a number of multi-center clinical trials and have gained regulatory approval for clinical application.^3,6,7^ However, the ability of EVLP to recondition marginal lungs is variable and depends on which criteria are used to select organs for perfusion before transplant. Published studies have shown variation in conversion rates from organs deemed unsuitable to those accepted, with the need remaining to standardize acceptance criteria.^8-10^ This remains the case despite evidence that utilization of extended-criteria donor organs does not necessarily alter posttransplant outcomes.^11-15^ It is therefore critical to develop a complete understanding of the immunological events that determine which lungs are amenable for EVLP reconditioning.

Influx of recipient neutrophils upon reperfusion mediates a number of proinflammatory sequelae that may facilitate acute lung injury, including production of damaging intermediates and cytokines, which culminates in damage and functional impairment of lung tissue.^16-20^ An aberrant neutrophil response is, therefore, undoubtedly at the heart of increased rates of posttransplant complications, including PGD. Previous studies have highlighted that one of the key mediators in facilitating lung dysfunction is interleukin-1 beta (IL-1β).^21,22^ This plays a key role in enhancement of neutrophil recruitment, a process at the heart of reperfusion injury.^22,23^ It, therefore, appears that parallels can be drawn between increased levels of IL-1β and neutrophil-mediated damage.

Previous work from our group has highlighted the importance of this pathway in determination of donor lung quality during EVLP before transplantation, with levels indicative of injury.^24^ IL-1β level after 30 min of EVLP was shown to be predictive of 1-y survival in a cohort of lungs that were subsequently transplanted.^25^ Perfusate samples from donor lungs in which the recipients did not survive 1 y demonstrated a greater propensity to stimulate neutrophil adhesion to endothelium in vitro via upregulation of adhesion molecules.^25^

This study aimed to improve our mechanistic understanding of the effects of IL-1β on donor lung function and evaluate its contribution to inflammation during EVLP and why this leads to poor outcomes after transplantation. Given the limited availability of research donor lungs and relative high cost of human EVLP research studies, it is imperative to develop methods that allow efficient use of resources. Here, we use an approach to split a pair of donor lungs and perform paired single-lung EVLP from the same donor, eliminating much inter-donor variability. This was used to assess effects of IL-1β on physiological organ function, pulmonary endothelial dysfunction, neutrophil adhesion in lung vasculature, and tissue gene expression, with perfusates additionally being used in in vitro activation assays.

## MATERIALS AND METHODS

### Research Approval and Ethics

This study was approved by the National Health Service North East Research Ethics Committee with reference number 16/NE/0230. It used donor lungs not accepted for transplantation and when consent from the donor next of kin was present for organ research. The study was approved by the National Health Service Blood and Transplant as Study 66: “Further Evaluation of EVLP to Improve Transplantation Outcomes.” Ethical approval to obtain blood from healthy volunteers for neutrophil isolation was granted by the Research Ethics Committee (12/NE/0121).

### Study Outline

**FIGURE 1.**
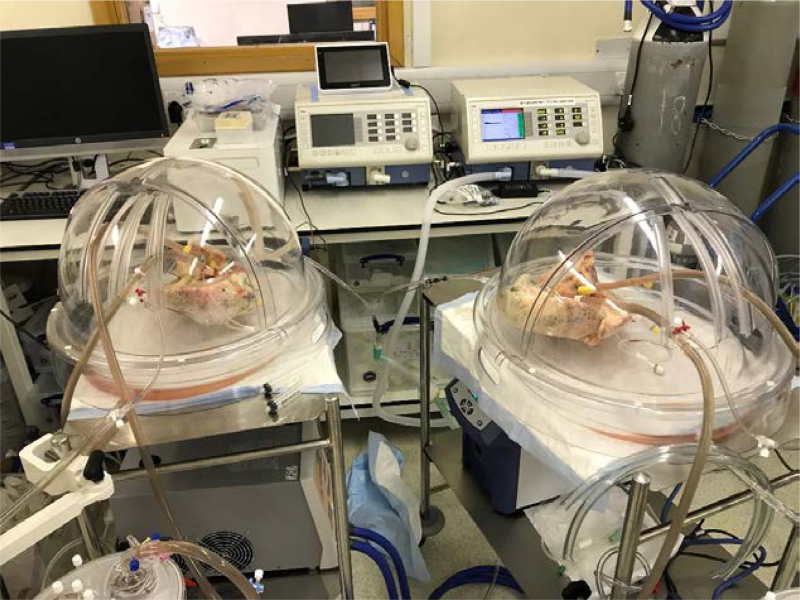
Dual EVLP circuits for lungs from the same donor, representative of split lung perfusions; individual lungs from the same donor were dissected and perfused simultaneously on separate Medtronic perfusion circuits. EVLP, ex vivo lung perfusion.

This study was conducted using a novel human split lung model where lungs from a single donor were perfused in 2, distinct single circuits (N = 4). Donor lungs declined for transplantation were dissected into individual right and left lungs before undergoing EVLP simultaneously on separate but identical perfusion circuits (Figure 1). Donor lungs were excluded on the basis of pathology that would likely compromise perfusion, with significant unilateral pathology on radiology or inspection also being criteria for exclusion. Rejection of donor lungs from our study occurred both before and following organ retrieval of lungs declined for clinical transplant. This approach minimized any influence of inter-individual variability between different donors. One lung from each pair was given a bolus of recombinant IL-1β sufficient to achieve a circuit concentration of 1 ng/ml before infusion of 4 × 10^7^ carboxyfluorescein succinimidyl ester (CFSE)-labeled neutrophils into both circuits. Numbers were quantified in real-time using flow cytometry. Tissue biopsies were analyzed for transcriptomic changes via Nanostring. Perfusate samples were used in functional in vitro assays. Lung pairs were compared individually before then being grouped into “control” and “treatment” groups for subsequent analysis.

### Lung Dissection Protocol

Lungs were procured in the same way as for clinical transplant by cardiothoracic transplant retrieval surgeons and preserved on ice during transportation. This was performed as part of the National Organ Retrieval Service in a standardized fashion. Once at the perfusion facility, lungs were dissected in preparation for EVLP on cold 0.9% saline. The common pulmonary artery (PA) was dissected at the bifurcation to isolate the left and right PA. XVIVO PA cannulae (XVIVO Perfusion AB, Sweden) were inserted into the left and right lung PA and secured with 2-0 silk sutures. The left atrium (LA) back wall was divided and trimmed to form circular cuffs around superior and inferior pulmonary veins (PVs) on both sides. The cuffs of XVIVO LA cannulae were prepared to match the sizes of the LA cuffs and sutured to them using 4-0 Prolene. The trachea was divided at the carina, deflating both lungs. The left and right main bronchi were then connected to shortened size 9 endobronchial tubes with 2-0 silk sutures. Care was taken to avoid blocking the right upper lobe bronchus. Individual lungs were weighed before EVLP. Sampling cannulae for acquiring perfusate samples were placed after the PV.

### EVLP Protocol

Lungs were perfused according to the Toronto EVLP protocol.^26^ A bespoke perfusate solution was used for our perfusions, which mimicked properties of well-established Steen solution. Briefly, a flow rate of 20% predicted cardiac output (CO) was calculated, which was initially used to perfuse the attached donor lungs. Flow rate was gradually increased to 40% CO with concurrent rewarming to 32°C, at which point ventilation was initiated. Tidal volume and respiratory rate were set based on perfusate temperature and ideal bodyweight of the donor. Once the perfusate reached 37°C, regular blood gas measurements were acquired to ensure parameters remained within physiological range throughout. The pH was maintained between 7.35 and 7.45, being adjusted with addition of Tris(hydroxymethyl)aminomethane solution if required. Airway measurements such as pressures and compliance were not able to be recorded for individual lungs because although lungs were perfused separately, they were ventilated via a shared ventilator.

Sufficient human recombinant IL-1β to achieve a concentration of 1 ng/ml (for 1.5 L of Steen use 1500 μl of 1 ng/μl IL-1β) was made up into 5 ml of sterile perfusate. This was infused as a bolus into one of the perfusion circuits with the other acting as a control. This approach was chosen to maximize the effects of cytokine infusion, thus allowing investigation of the mechanisms underlying the previously shown association between higher IL-1β concentrations in perfusate during EVLP and poorer clinical transplant outcomes.

Upon completion of 3 h of EVLP, lungs were detached, drained of all intravascular perfusion fluid, and weighed. Biopsies were acquired from the center of each lobe. Large airways were avoided. Samples were placed in RNAlater and stored at 4°C for 24 h and then transferred to −80°C for long-term storage.

### Leukocyte Tracking Assay

Neutrophils were isolated from healthy blood donors using a MACSXpress Whole Blood Isolation Kit (Miltenyi Biotec) as per manufacturer’s instructions and resuspended to a density of 5 × 10^6^ cells/ml. Isolated neutrophils were labeled with Celltracker CFSE (Biolegend) as per manufacturer’s instructions. 4 × 10^7^ neutrophils were simultaneously infused into each perfusion circuit gradually over 1 min through the arterial line. A small amount of perfusate was drawn up before infusing into the circuit, ensuring no air was pushed into the lung vasculature. Infusion took place exactly 60 min after infusion of IL-1β. Perfusates were acquired throughout perfusion. Following EVLP, lungs were detached from the circuits and a leukocyte filter was attached to PA and PV cannulae. The pump was left on for 5 min. Filters were washed with phosphate-buffered saline in an anterograde direction before multiple retrograde washes were performed to remove captured leukocytes.

Collected perfusate samples were centrifuged at 200*g* for 5 min, with 4 ml of each sample aliquoted into cryovials and frozen at −80°C for subsequent analysis. The cell pellet was washed and resuspended in PBS with 2% fetal bovine serum. Ten microliters of resuspended cells was run through a FACS Accurri C6 flow cytometer and multiplied up to give total cell counts for each perfusate sample. Results were plotted using Prism 8 software (GraphPad Software, CA). All final counts were adjusted to total lung capacity (TLC).

### Enzyme-linked Immunosorbent Assay

An enzyme-linked immunosorbent assay was used to measure concentration of soluble intercellular adhesion molecule-1 (sICAM-1) (R&D Systems, cat. no DY720-05) and von-Willebrand factor (vWF) (R&D Systems, cat. no DY2764-05) from perfusate samples. Standards for each plate were used to generate a curve. Perfusates were diluted to 1:1000 for sICAM-1 and used neat for vWF. Standards and perfusate samples were applied in technical duplicate. Optical density of plates was assessed through the Synergy spectrophotometer and Gen5 plate reading software (BioTek) at 450 nm. Final concentration values were adjusted to TLC.

### Microfluidic Flow Assay

Perfusate stimulation of neutrophil-endothelium adhesion was assessed using a microfluidic platform (Cellix Ltd). Vena8 Endothelial + Biochips were coated with 8–10 μl of fibronectin (100 μg/ml) and incubated overnight at 4°C. Human pulmonary microvascular endothelial cells (HPMECs) (PromoCell GmbH) were seeded onto the chips at 1.5 × 10^7^/ml and left to attach for 15 min. Forty microliters of treatments were added to each well per channel with HPMECs then incubated at 37°C/5% CO_2_ for 4 h. For blocking experiments, perfusates were preincubated with an IL-1β neutralizing antibody (NAb) (R&D Systems) at 4 μg/ml for 30 min prior. Neutrophils were isolated from healthy volunteers as previously detailed. 1 × 10^7^ cells were collected in Hank’s balanced salt solution (HBSS) and left at room temperature for 20 min. These were stained with 20 μM Celltracker CFSE (Biolegend) in 2 ml of cold PBS (no Ca^2+^, no Mg^2+^) at 4°C in the dark for 10 min before 8 ml of complete Roswell Park Memorial Institute media was added to quench the reaction. Cells were washed twice in complete Roswell Park Memorial Institute before being resuspended to a density of 1 × 10^6^/ml.

The microfluidic assay was run with a Mirus Evo Nanopump controlled via PC link with VenaFluxAssay software (Cellix Ltd). Labeled neutrophils were allowed to flow over the endothelial layer for 3.5 min before images were acquired at points 2, 3, 4, 5, and 6 on the biochip. Images were analyzed and quantified for neutrophil adhesion via DucoCell software. Results were plotted in excel and presented using Prism 8 software (GraphPad).

### Flow Cytometry Analysis

HPMECs were seeded onto 24-well plates and stimulated with perfusates for 4 h. In the case of blocking experiments, these were preincubated with an IL-1β NAb (R&D Systems) at 4 μg/ml for 30 min prior. Cells were removed from the plates with Accutase before being washed and resuspended in cold FACS buffer (PBS, 2% fetal bovine serum) with flow cytometry antibodies for 30 min at 4°C. This panel consisted of directly conjugated antibodies specific for PECAM-1, ICAM-1, vascular cell adhesion molecule-1 (VCAM-1), and E-Selectin. These were washed twice more in cold FACS buffer before being run on a BD FACSCanto II using FACSDiva software. Compensation was performed using OneComp eBeads (eBioscience).

### RNA Isolation

Tissue samples collected from EVLP were stored in RNAlater at −80°C until required. Twenty-microgram sections were dissected and homogenized using a Tissue Lyser II for 2 min in RLT Plus buffer with stainless steel beads (Qiagen). RNA was isolated using the RNeasy Mini Plus Kit (Qiagen) as per the manufacturer’s instructions. Purified RNA was analyzed using a Nanodrop One for A280/260 and A260/230 ratios before being quantified for RNA concentration precisely using a Qubit high-sensitivity RNA assay. Biopsies were prepared and analyzed individually.

### RNA Profiling

Nanostring technology was used to perform targeted RNA investigation. Analysis of homogenized tissue was performed using a nCounter FLEX analysis system with the nCounter Human immunology v2 Panel. Biopsies were analyzed individually, then collated into control and stimulated groups. Results from profiling experiments were collated and processed using nSolver software (NanoString). The panel contained 6 positive controls, 8 negative controls, and 15 internal reference genes. RNA count from each stimulated lung was compared to the corresponding control lung from each pair before relative changes were compared between different lung cohorts.

### Statistical Analysis

A TLC correction factor was applied to measurements of neutrophils and inflammatory mediators obtained from the perfusate samples to correct for differences in lung size within lung pairs. The correction factor was applied to each donor with an adjustment factor of 0.45 for left lungs and 0.55 for right lungs to account for the greater surface area of the latter group during EVLP. The predicted TLC calculation was performed using standard formulae based on donor sex and height.^27^

All error bars on graphs are representative of SEM. For comparison of control and treatment groups paired t tests were used. For the RNA profiling work, ratio-paired t tests were chosen because donors had very different levels of RNA for many genes assessed in our panel. It was felt that using the ratio of difference offered more of a valid comparison. For statistical tests, *P* values below 0.05 were considered statistically significant. * Denotes values as follows: *P < 0.05; **P < 0.01; ***P < 0.001; ****P < 0.0001.

## RESULTS

### Lung Donor and Perfusion Characteristics

Four donor lung pairs that declined for transplantation were included in this study. Table 1 highlights key donor information, with lungs used for this study declined on the basis of poor function or bilateral consolidation before retrieval. Table 2 highlights ischemic times and weight gain over the course of EVLP.

**TABLE 1.** Donor data for lungs used in the split lung model

Perfusion	Donor age/sex	Smoker?	Previous lung disease?	Bodyweight (kg)/height (cm)	Donation	Cause of death	Approx. duration of ventilation (h)	Last pO_2_ (kPa)	CXR findings	Reason for decline
Donor 1	56 F	No	No	80/165	DBD	ICH	48	44.3	Unknown	Inspection
Donor 2	22 M	Yes	No	65/180	DCD	Asphyxiation	132	51.2	Infiltrates RL	Inspection
Donor 3	59 M	No	No	95/171	DBD	ICH	48	37.7	Lungs clear	Function
Donor 4	47 F	No	No	50/171	DBD	ICH	72	12.2	Lungs clear	Function

CXR, chest x-ray; DBD, donation after brain death; DCD, donation after circulatory death; ICH, intracerebral hemorrhage; pO2, partial pressure of oxygen; RLL, right lower lobe; RML, right middle lobe.

**TABLE 2.** Perfusion data for lungs used in the split lung model

Perfusion	Right/left	CIT (h, min)	+ IL-1β? (Y/N)	Weight pre	Weight post
Donor 1	Right	10, 30	N	593.5	1040
Donor 1	Left	10, 30	Y	408	1064.5
Donor 2	Left	13, 40	N	666.5	1064.5
Donor 2	Right	13, 40	Y	846	1769.5
Donor 3	Left	17, 10	N	424.5	1059
Donor 3	Right	17, 10	Y	557.5	1295
Donor 4	Left	4, 37	N	366	467
Donor 4	Right	4, 37	Y	484.5	923.5

CIT, cold ischemic time; IL-1β, interleukin-1β; Y/N, Yes/No.

### Circulating Numbers of Neutrophils Ex Vivo Are Lower in Perfusates of Lungs Stimulated With IL-1β

**FIGURE 2.**
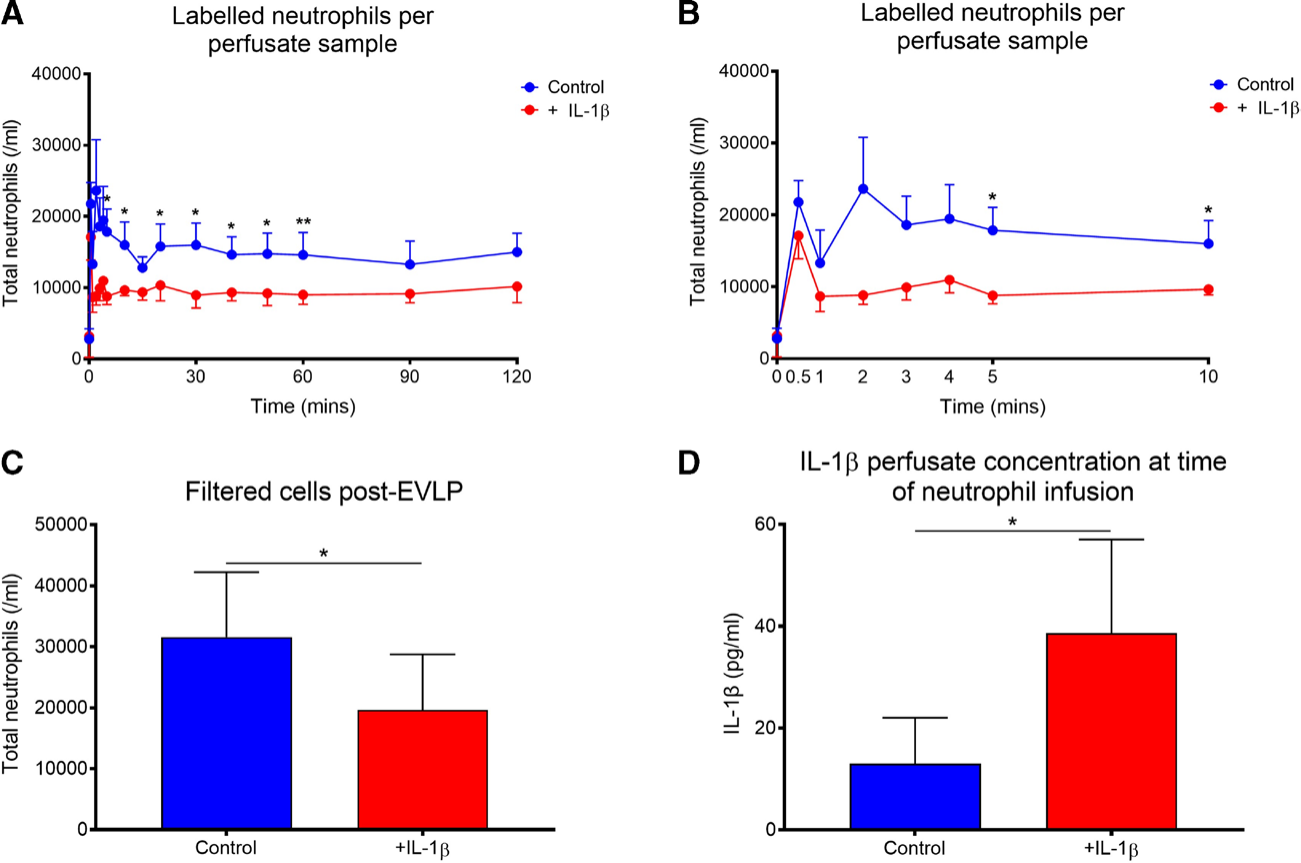
Labeled neutrophils in perfusate samples. Whole lungs were dissected and split into individual lungs and perfused ex vivo, with control (N = 4) and IL-1β-stimulated (N = 4). CFSE-labeled neutrophils were infused as a bolus into “T0,” with regular perfusate samples acquired and then analyzed for FITC + events via an Accuri C6 flow cytometer. Cell numbers were normalized to TLC and then plotted using Prism 8 software. These numbers are presented as total time (A) and the initial 10 min following cell infusion (B). This achieved statistical significance at several time points (paired t test). Upon completion of perfusion, lungs were detached and any remaining “free” perfusate was filtered for labeled leukocytes before being measured for cells in the same way (C) (*P* = 0.042). Perfusate concentration of IL-1β was significantly different between control and stimulated groups at the point of neutrophil infusion into the circuits (*P* = 0.016) (ratio paired t test). N = 4. CFSE, carboxyfluorescein succinimidyl ester; EVLP, ex vivo lung perfusion; FITC, fluorescein isothiocyanate; IL-1β, interleukin-1β; TLC, total lung capacity.

Lung pairs that declined for transplant were dissected into individual lungs and perfused simultaneously (N = 4). Perfusate samples acquired over 120 min of perfusion were quantified for CFSE+ neutrophils. Consistently lower numbers of neutrophils were observed in lungs stimulated with IL-1β, which achieved significance at 5-, 10-, 15-, 30-, 40-, 50-, and 60-min following infusion (Figure 2A and B). When remaining perfusate was filtered post-EVLP, significantly fewer cells were observed in perfusate of IL-1β-stimulated lungs following EVLP (*P* = 0.042) (Figure 2C). IL-1β concentration was significantly different between groups at the point of neutrophil infusion (*P* = 0.016) (Figure 2D).

### IL-1β Stimulation Accentuates Weight Gain in Lungs and Endothelial Dysfunction Ex Vivo

**FIGURE 3.**
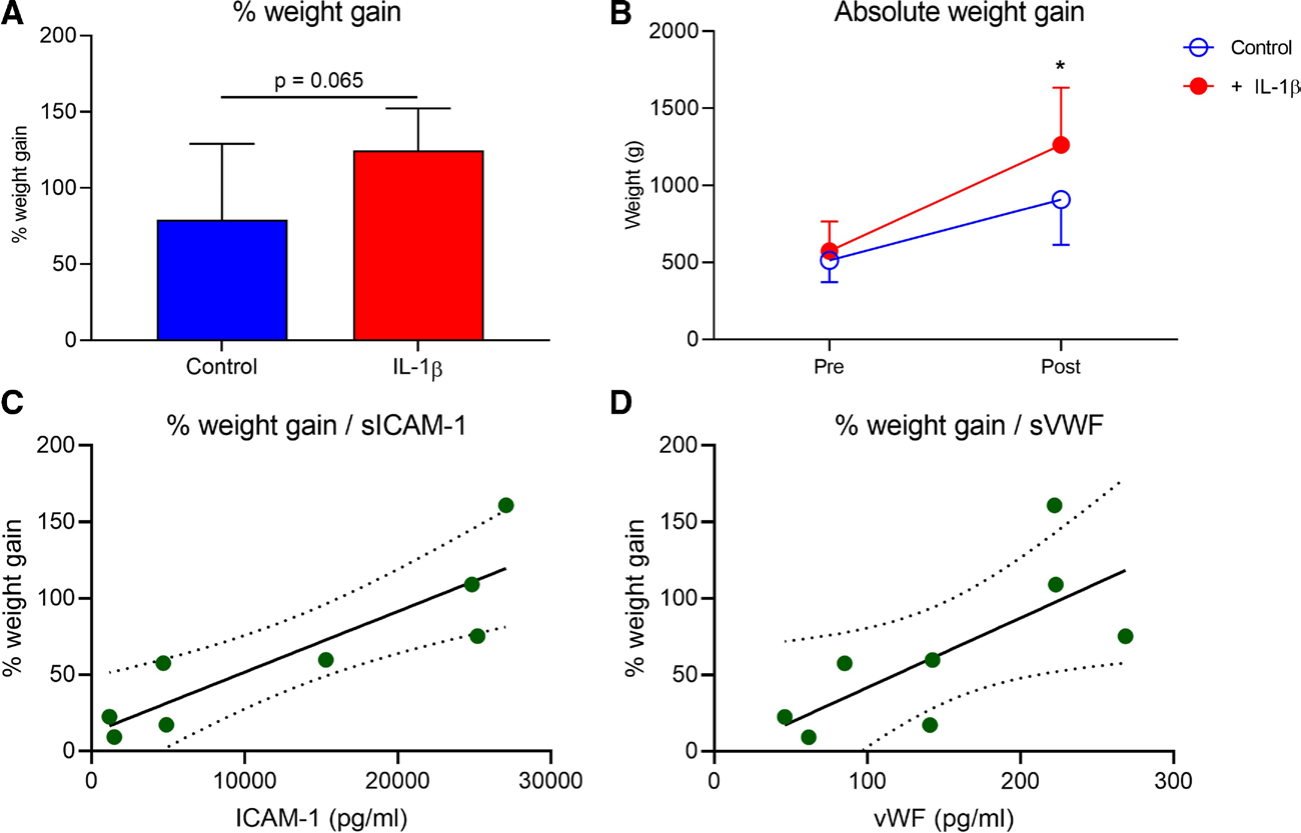
Weight gain is increased by IL-1β and correlates with endothelial dysfunction. Whole lungs were dissected and split into individual lungs and perfused ex vivo, with control and IL-1β-stimulated. Lung weight was measured during both preperfusion and postperfusion, and the change is presented as both percentage weight gain (A) and absolute weight gain (B). Lungs stimulated with IL-1β gained more weight than control lungs when shown as both a percentage increase (*P* = 0.065) and absolute value (*P* = 0.046). Concentration of sICAM-1 and vWF were measured in perfusate samples taken after 120 min of EVLP and then adjusted to TLC (pg/ml). A linear regression model was then used to model the relationship between concentration and edema formation ex vivo. Weight gain (%) correlated significantly with sICAM-1 (R^2^ = 0.71, *P* = 0.0043) (C) and vWF (R^2^ = 0.390, *P* = 0.040) (D) highlighting the relationship between edema and endothelial cell activation. N = 4. EVLP, ex vivo lung perfusion; IL-1β, interleukin-1β; sICAM-1, soluble intercellular adhesion molecule-1; TLC, total lung capacity; vWF, von-Willebrand factor.

Weight gain during EVLP was chosen as a surrogate marker for alveolar and interstitial edema formation. Lungs from each pair stimulated with IL-1β showed greater weight gain over the course of EVLP as both percentage change (*P* = 0.065) (Figure 3A) and absolute increase (*P* = 0.046) (Figure 3B). sICAM-1 and vWF were quantified in perfusates using standard enzyme-linked immunosorbent assay. Final concentrations were normalized to donor lung TLC (pg/ml) and then compared against percentage weight gain in a linear regression model. Edema correlated significantly with sICAM-1 (R^2^ = 0.71, *P* = 0.0043) and vWF (R^2^ = 0.39, *P* = 0.040) (Figure 3C and D).

### IL-1β Stimulation Does Not Significantly Affect Lung Physiology During EVLP

**FIGURE 4.**
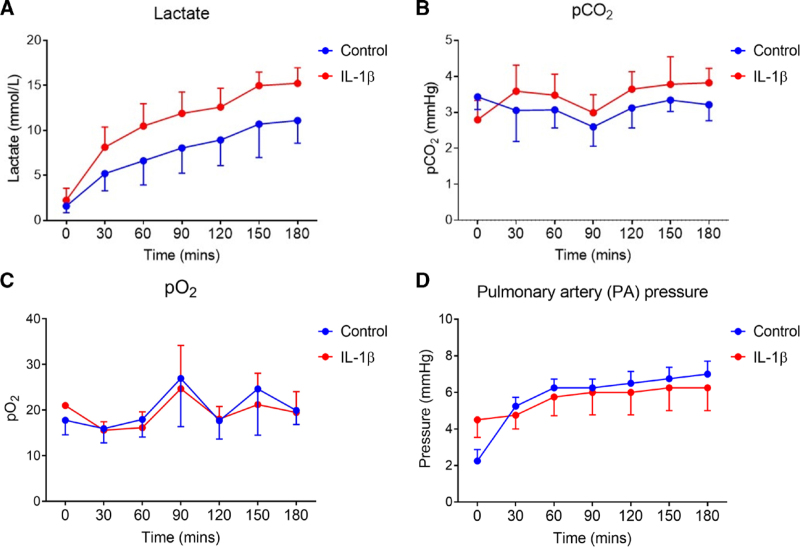
Physiology is not significantly altered via infusion of IL-1β during EVLP. pCO_2_, pO_2_, and lactate were measured during EVLP at regular time points throughout perfusions. PAP was measured via a pressure transducer attached to the inflow cuff of the EVLP circuit. Lactate increased in all lungs during perfusion, with this increase greater in stimulated lungs (A). pCO_2_ was seen to be generally higher in stimulated lungs throughout EVLP (B). For pO_2_, this did not vary significantly between control and stimulated lungs (C). PAP did not vary between control and stimulated groups (D). N = 4. EVLP, ex vivo lung perfusion; IL-1β, interleukin-1β; PAP, pulmonary artery pressure; pCO_2_, partial pressure of carbon dioxide; pO_2_, partial pressure of oxygen.

Lactate level, partial pressure of oxygen, and partial pressure of carbon dioxide (pCO_2_) were plotted over the course of split lung perfusion experiments via blood gas measurements. PA pressure was measured by attachment of a pressure monitor to the arterial cuff of the EVLP circuit. Lactate and pCO_2_ were generally higher in stimulated lungs relative to controls (Figure 4A and B). Partial pressure of oxygen and PA pressure were not altered by IL-1β infusion during EVLP (Figure 4C and D).

### Gene Expression Is Differentially Regulated by IL-1β

**FIGURE 5.**
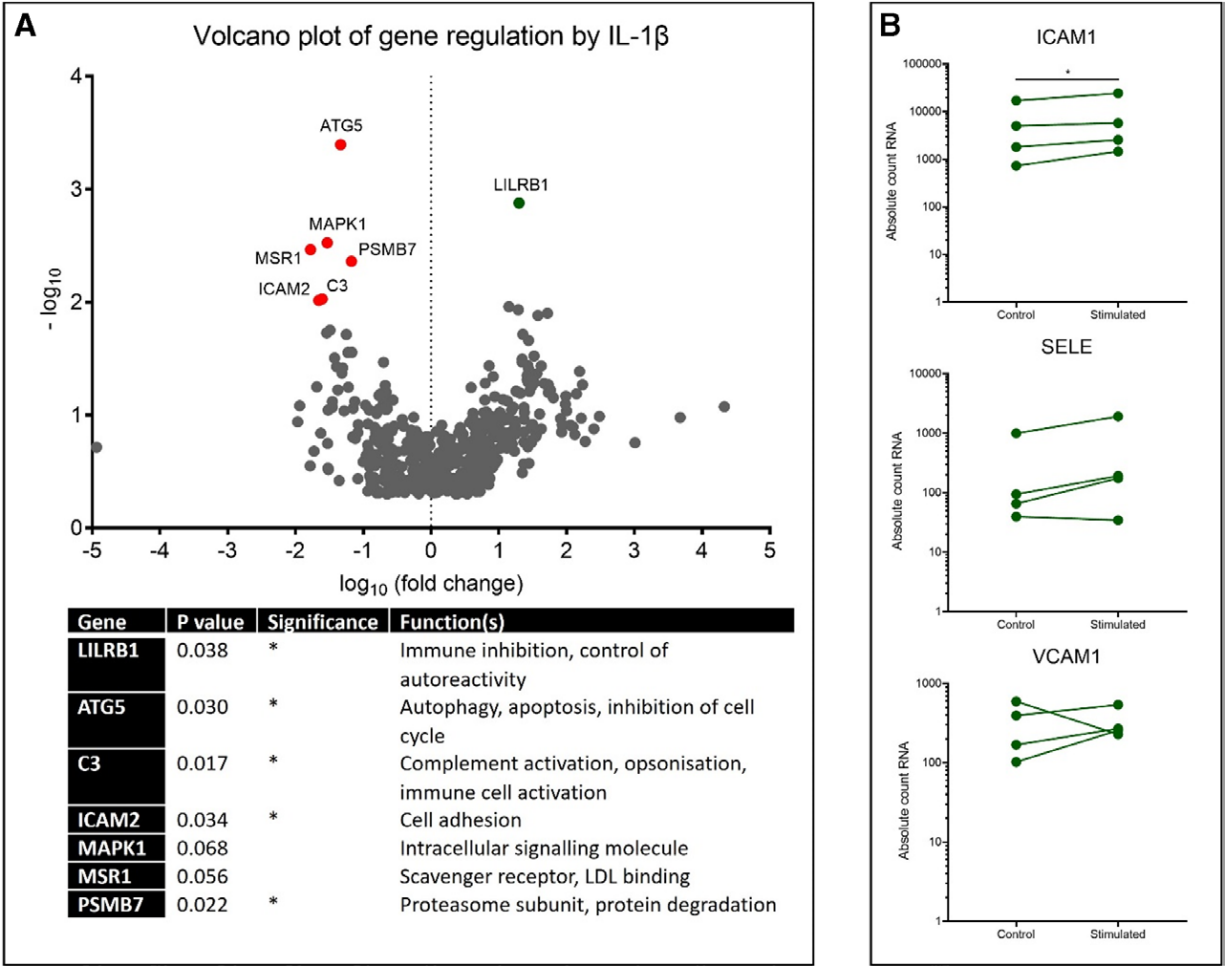
Differential expression of genes altered by IL-1β stimulation. Genes involved in a variety of cellular functions and processes were altered by way of IL-1β during EVLP of donor lungs. The volcano plot highlights genes significantly altered in transcription level within our cohort (A). LILRB1 was significantly upregulated (*P* = 0.038), whereas expression of ATG5 (*P* = 0.030), C3 (*P* = 0.017), ICAM-2 (*P* = 0.034), and PSMB7 (*P* = 0.022) were significantly downregulated (A). Downregulation of MAPK1 and MSR1 did not achieve statistical significance. We specifically examined the genes encoding ICAM-1, E-selectin, and VCAM-1, with ICAM-1 (*P* = 0.021) and SELE (*P* = 0.059) upregulated by infusion of IL-1β into our system (B). ATG5, autophagy-related gene 5; EVLP, ex vivo lung perfusion; ICAM-2, soluble intercellular adhesion molecule-2; IL-1β, interleukin-1β; LILRB1, leukocyte immunoglobulin-like receptor subfamily B member 1; Mapk1, mitogen-activated protein kinase 1; MSR1, macrophage scavenger receptor 1; VCAM-1, vascular cell adhesion molecule-1.

Transcriptomic effects of IL-1β were investigated in tissue-acquired post-EVLP. The volcano plot highlights genes that were altered significantly by infusion of IL-1β and achieved at least a 2x fold-change (Figure 5A). Leukocyte immunoglobulin-like receptor subfamily B member 1, involved in immunoregulation, was significantly upregulated (*P* = 0.038), whereas expression of autophagy-related gene 5 (*P* = 0.030), complement (C3) (*P* = 0.017), ICAM2 (*P* = 0.034), and PSMB7 (*P* = 0.022) were significantly downregulated. Decreases of mitogen-activated protein kinase 1 and macrophage scavenger receptor 1 were not statistically significant. We specifically examined genes encoding expression of ICAM-1, E-selectin, and VCAM-1 (Figure 5B). Expression of ICAM-1 messenger RNA (mRNA) significantly increased in IL-1β-stimulated lungs (*P* = 0.021), with expression of E-selectin showing a trend towards an increase (*P* = 0.059). VCAM-1 expression was not altered. For ICAM-1 and E-selectin, increase is seen most noticeably in one lung pair, with the others increasing to a lesser extent.

### Perfusates From Lungs Stimulated With IL-1β Confer Greater Neutrophil Adhesion to Conditioned Endothelial Cells In Vitro

**FIGURE 6.**
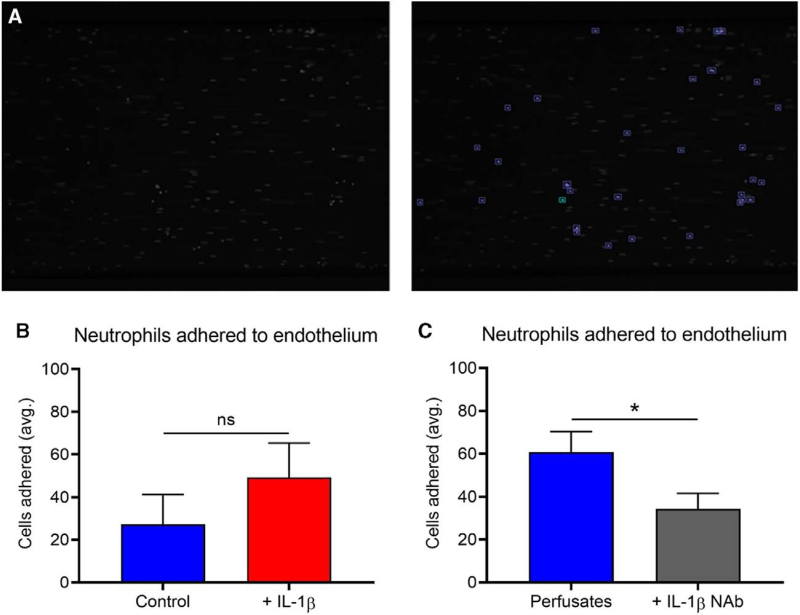
Adhesion of labeled neutrophils to perfusate-conditioned endothelial cells. A microfluidic flow assay was used to quantify neutrophil adherence to endothelium under flow. Bound cells were quantified with unbound cells discounted from analysis following completion of the assay. Adhered cells are pictured before and following analysis (A). Perfusates taken from IL-1β-stimulated lungs (N = 4) was compared to control lung perfusates (N = 4) for their ability to facilitate neutrophil adhesion to conditioned pulmonary endothelial cells in vitro (B). Samples used from IL-1β-stimulated lungs conferred a greater degree of adhesion onto endothelial cells. When all perfusates (N = 8) were preincubated for 30 min with an IL-1β NAb, this reduced adhesion significantly (*P* = 0.025) (C). IL-1β, interleukin-1β; NAb, neutralizing antibody.

Perfusates taken at 120 min were used to stimulate CFSE-labeled neutrophil adhesion to HPMECs precoated onto specialized microfluidic chambers in vitro for 4 h. Perfusates from stimulated lungs (N = 4) conferred greater adhesion of neutrophils to endothelium in vitro, as measured by greater numbers of cells per image field (Figure 6B). When all perfusates (N = 8) were preincubated with an IL-1β NAb at 4 μg/ml for 30 min, this significantly downregulated adhesion relative to controls (*P* = 0.025) (Figure 6C).

### Adhesion Marker Expression of Conditioned Endothelial Cells In Vitro

**FIGURE 7.**
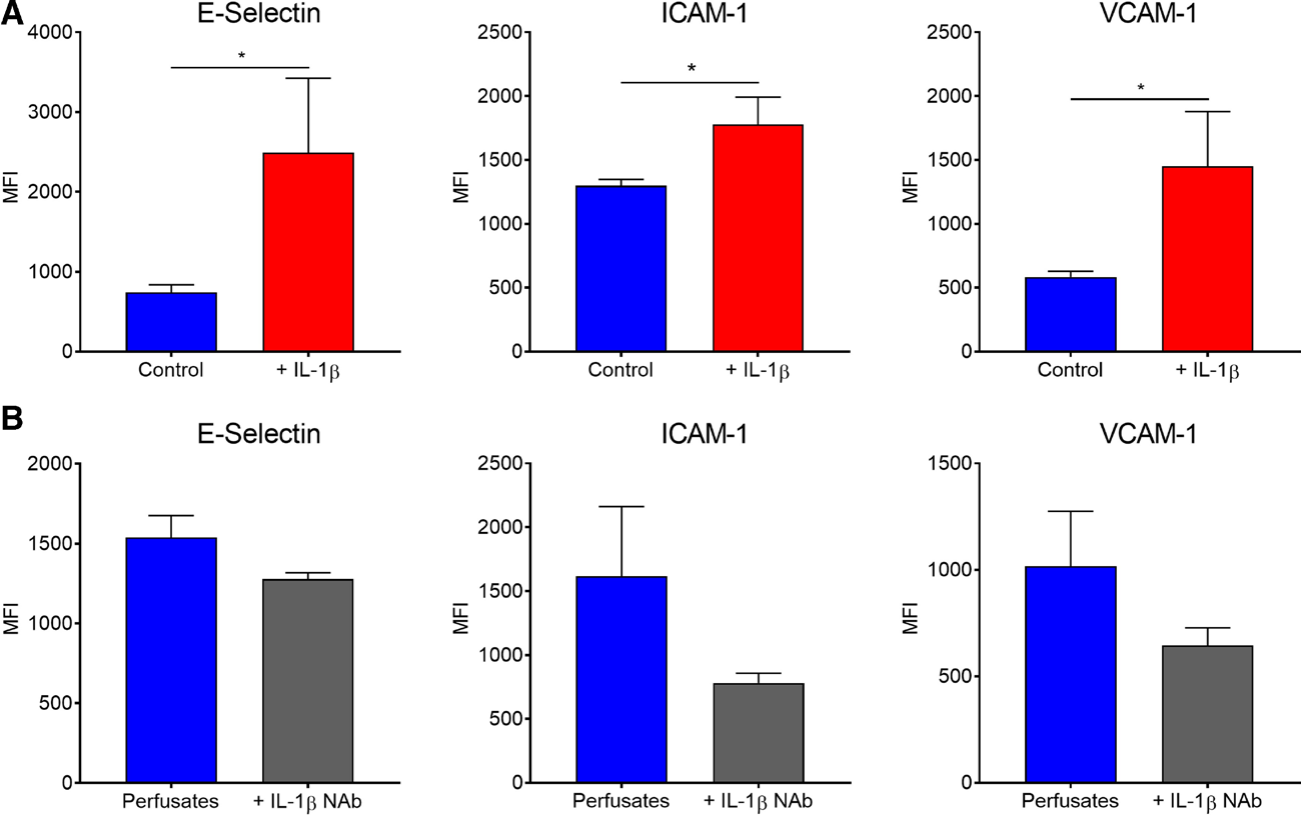
Expression of adhesion markers on perfusate-conditioned endothelial cells. Perfusates taken from IL-1β-stimulated lungs (N = 4) were compared to control lung perfusates (N = 4) for their ability to induce expression of 3 different adhesion markers. E-Selectin (*P* = 0.027), ICAM-1 (*P* = 0.027), and VCAM-1 (*P* = 0.046) were all upregulated to a greater degree on the surface of pulmonary endothelial cells when stimulated lung perfusates were used (A). When all perfusates (N = 8) were preincubated for 30 min with an IL-1β NAb, this reduced expression of E-Selectin (13%), ICAM-1 (20%), and VCAM-1 (15%) (B). ICAM-1, intercellular adhesion molecule-1; IL-1β, interleukin-1β; NAb, neutralizing antibodies; VCAM-1, vascular cell adhesion molecule-1.

HPMECs were analyzed for E-selectin, ICAM-1, and VCAM-1 expression via flow cytometry following 120 min of perfusate stimulation. Perfusates from IL-1β-stimulated lungs (N = 4) induced greater upregulation of E-selectin (*P* = 0.038), ICAM-1 (*P* = 0.071), and VCAM-1 (*P* = 0.069) than control lungs (N = 4) (Figure 7A). When all perfusates (N = 8) were preincubated with an IL-1β NAb at 4 μg/ml for 30 min, this demonstrated a trend of downregulating expression of all markers (Figure 7B). Median fluorescence intensity is higher in “perfusates” for group B, as this represents combined values for all perfusates (N = 8) rather than the unstimulated group (N = 4).

## DISCUSSION

Measurement of perfusate biomarkers during EVLP could provide an objective measurement of organ suitability for transplantation, via stratification on the basis of inflammation or injury-associated proteins or gene signatures.^28-30^ The IL-1 pathway has been implicated in multiple lung pathologies, with data linking IL-1β level with both inflammatory and fibrotic diseases, such as acute lung injury^31,32^ and idiopathic pulmonary fibrosis.^33,34^

Previous work from our group has demonstrated association between IL-1β concentrations in perfusate during EVLP and medium-term posttransplant outcomes. In this study, we used an approach to preclinical EVLP whereby right and left lungs from the same donor were separated and perfused simultaneously but independently on identical circuits. Furthermore, utilization of healthy donor neutrophils replicated reperfusion following transplantation. It is our belief this approach is not only beneficial from an experimental design perspective but also maximizes the impact of precious research organs. Although IL-1β in the context of lung injury is well-defined, the scope of this work was to evaluate its role within a human model of EVLP, which we felt would enhance many of these previous observations and provide a robust framework by which to do so.

Our study showed that priming a donor lung with a bolus of IL-1β significantly reduced numbers of exogenously administrated neutrophils while increasing pulmonary edema and pulmonary endothelial activation compared to controls. The bolus used generated a perfusate concentration equivalent to that seen from spontaneous IL-1β production in lungs during EVLP that performed poorly posttransplant in the DEVELOP-UK study (Figure 2D).^28,35^ Perfusates collected from IL-1β-stimulated lungs conferred greater endothelial activation during in vitro studies, as highlighted via our microfluidic adhesion assay and flow cytometry analyses of adhesion molecules. Evaluation of lung tissue post-EVLP highlighted that expression of ICAM-1 mRNA was significantly higher in lungs stimulated with IL-1β. The observations made by Andreasson et al, identified that perfusate IL-1β concentration just 30 min into EVLP was a predictor of 1-y survival following lung transplantation.^25^ We feel the work performed here provides valuable insights into the mechanisms by which IL-1β is acting during EVLP and suggests the associations reported by Andreasson et al are causal. Our results also support the findings of Ferdinand et al, who recently showed that inflammasome-related gene expression increased in lungs that were declined for transplant following EVLP.^36^

Disruption of endothelial integrity is a hallmark of pulmonary insult.^37^ We observed that sICAM-1 and vWF, associated with endothelial dysfunction, correlated with extent of pulmonary edema defined by percentage weight gain. Previous literature has identified a link between sICAM-1 and PGD risk, which also remains the case following EVLP.^38,39^ Correlation between endothelial activation and edema in our study was strongest for sICAM-1, which supports these observations that this relates to the extent of lung injury following EVLP.^39^ Although there were trends toward increasing lactate and pCO_2_ in IL-1β-stimulated lungs, these did not achieve statistical significance.

To complement our findings using protein biomarkers, targeted mRNA analysis of the lung tissue was performed. Transcription of a number of genes involved in processes such as protection of cellular degradation (autophagy-related gene 5) and immune regulation (PSMB7) was differentially regulated by IL-1β. We also found that expression of ICAM-1 mRNA was significantly upregulated after 3 h of stimulation. These data further support literature that IL-1β provokes greater leukocyte binding and disruption of the endothelium during reperfusion injury.^25^

We used in vitro assays to confirm the ex vivo observations, namely that IL-1β reduced circulating neutrophils and facilitated endothelial damage. When HPMECs were exposed to perfusates from stimulated lungs, this facilitated greater neutrophil adhesion, which was abolished when we preincubated perfusates with an IL-1β NAb (*P* = 0.015). Expression of adhesion markers increased when we used stimulated lung perfusates, which was similarly downregulated when preincubated with an IL-1β NAb. These data support conclusions made in the other parts of our study, underlining how IL-1β mediates neutrophil-endothelial adhesion, outlined by Andreasson et al.^25^

A major strength of our study was the EVLP model using split lung pairs from the same donor, perfused in real-time on separate circuits. This approach allowed us to control for the inherent clinical differences between different donors that are confounders in experimental EVLP studies that perfuse lung pairs, such as prolonged ischemic times in some of our study participants. Additionally, using neutrophils from a different, allogeneic donor more accurately replicates lung reperfusion in vivo, mimicking the reality of transplantation. Although circulating neutrophil numbers in our model (3–5 × 10^6^/ml) fall below those observed in vivo, the aim was to investigate neutrophil kinetics, that is, adhesion during lung injury, rather than quantify damage caused by their activation ex vivo.

One modification to our model could be to extend the duration of IL-1β stimulation before neutrophil infusion into the circuits. This would likely increase the gap between the 2 populations, enabling a more precise impact of therapeutic supplementation on circulating cell numbers. This may provide more time to observe variations in gene and protein expression. However, in the work performed by Ferdinand et al (2021), tangible changes in gene expression were observed after 2–4 h of EVLP, highlighting that the duration of our perfusions provided ample time for transcriptomic changes to occur.^36^ This latter point, along with extensive injury conferred by infusion of exogenous IL-1β, is the basis for our decision to perfuse lungs in our study for a relatively short duration of 3 h.

Another future pathway for this work would be to block endogenous IL-1β within the perfusate and lung tissue before neutrophil infusion. This would be done to ascertain whether expected decreases in edema and inflammation would be observed, with our expectation being that blockade would reproduce observations made by Andreasson et al in an in vitro setting.^25^ It is this work that would be key in ascertaining whether or not cytokine blockade before transplantation is able to render marginal lungs suitable for transplantation. One means of doing this could be to use an anti-IL-1β antibody such as Anakinra, which has already been demonstrated to be effective in reducing rates of heart failure in cases of myocardial infarction, while not significantly increasing infection rates.^40,41^ We feel that EVLP provides an ideal means to administer therapeutic modulation before transplantation in the future.

A potential weakness of our work is the relatively small sample size. After eliminating lungs with significant unilateral pathology, we used 4 donor lung pairs which using our novel model equates to n = 8 lungs studied as part of assays to investigate functions of IL-1β during EVLP. However, other work using real-time organ perfusion has used comparable numbers to our work here because human whole organ-based research must use a valuable and limited resource.^36,42^

In summary, we used a human split donor lungs model of EVLP, allowing for robust inbuilt controls for experimental intervention in each donor. We used this model to highlight IL-1β as a key modulator of neutrophil adhesion to pulmonary vasculature, as well as endothelial activation and disruption. An obvious next step would be testing if blockade of endogenous IL-1β during EVLP would maintain endothelial integrity and reduce adhesion molecule expression before transplantation, decreasing rate of detrimental posttransplant outcomes.

## ACKNOWLEDGMENTS

The authors acknowledge the use of Servier Medical Art (smart.servier.com, accessed January 2023) for part of the generation of the visual abstract presented as part of this article.
